# Specific growth rate governs *AOX1* gene expression, affecting the production kinetics of *Pichia pastoris* (*Komagataella phaffii*) P_*AOX1*_-driven recombinant producer strains with different target gene dosage

**DOI:** 10.1186/s12934-019-1240-8

**Published:** 2019-11-01

**Authors:** Javier Garrigós-Martínez, Miguel Angel Nieto-Taype, Arnau Gasset-Franch, José Luis Montesinos-Seguí, Xavier Garcia-Ortega, Francisco Valero

**Affiliations:** Department of Chemical, Biological and Environmental Engineering, School of Engineering, Universitat Autònoma de Barcelona, 08193 Bellaterra (Cerdanyola del Vallès), Spain

**Keywords:** *AOX1* promoter, Heterologous gene dosage, Transcription analysis, *Pichia pastoris*, Gene expression/regulation, *MIT1*, Specific growth rate influence, Operational mode

## Abstract

**Background:**

The P_*AOX1*_-based expression system is the most widely used for producing recombinant proteins in the methylotrophic yeast *Pichia pastoris* (*Komagataella phaffii*). Despite relevant recent advances in regulation of the methanol utilization (MUT) pathway have been made, the role of specific growth rate (*µ*) in *AOX1* regulation remains unknown, and therefore, its impact on protein production kinetics is still unclear.

**Results:**

The influence of heterologous gene dosage, and both, operational mode and strategy, on culture physiological state was studied by cultivating the two P_*AOX1*_-driven *Candida rugosa* lipase 1 (Crl1) producer clones. Specifically, a clone integrating a single expression cassette of *CRL1* was compared with one containing three cassettes over broad dilution rate and *µ* ranges in both chemostat and fed-batch cultivations. Chemostat cultivations allowed to establish the impact of *µ* on the MUT-related *MIT1* pool which leads to a bell-shaped relationship between *µ* and P_*AOX1*_-driven gene expression, influencing directly Crl1 production kinetics. Also, chemostat and fed-batch cultivations exposed the favorable effects of increasing the *CRL1* gene dosage (up to 2.4 fold in *q*_*p*_) on Crl1 production with no significant detrimental effects on physiological capabilities.

**Conclusions:**

P_*AOX1*_-driven gene expression and Crl1 production kinetics in *P. pastoris* were successfully correlated with *µ*. In fact, *µ* governs MUT-related *MIT1* amount that triggers P_*AOX1*_-driven gene expression—heterologous genes included—, thus directly influencing the production kinetics of recombinant protein.

## Background

In the last two decades, *Komagataella phaffii*, which was formerly known as *Pichia pastoris*, has emerged as a promising host for recombinant protein production (RPP) [1–6]. Also, it has lately been increasingly used for metabolite production. Mattanovich et al. have summarized the main uses of *P. pastoris* for metabolite production [7]. The potential of *P. pastoris* for hosting the production of recombinant proteins is increased by its ability to grow at high cell densities (ca. 100 g L^−1^ dry cell weight) on defined media, the availability of strong protein expression systems, the possibility to secrete the target proteins to the extracellular medium, its enabling eukaryotic post-translational modifications [8, 9] and a reference genome sequence [10].

The alcohol oxidase 1 promoter (P_*AOX1*_) expression system has been widely used for recombinant protein production on *P. pastoris*. In terms of regulation, P_*AOX1*_ is strongly inducible by methanol and repressible by both glucose and glycerol. Its tight regulation allows bioprocess decoupling into a first phase of biomass generation and a second phase of where heterologous gene expression is induced by the addition of methanol. Properly designing the induction phase is crucial to obtain acceptable amounts of recombinant protein [2, 6, 11, 12]. P_*AOX1*_ typically allows large amounts of proteins to be obtained [3, 13–15]; however, the need to use methanol leads to some drawbacks related to plant safety, high oxygen consumption and also high heat production [16, 17].

In the literature, recent relevant advances in P_*AOX1*_ regulation can be found [3]. Thus, promoter sequence analysis has allowed several binding sites for transcription factors (TFs) to be identified. Most such TF were previously known and have been related to stress response, glucose repression and oxygen consumption [18]. Three of them (Mig1, Mig2 and Nrg1) have emerged as strong repressors of genes involved in methanol uptake [19], whereas three others (Mxr1, Mit1 and Prm1) have proved crucial triggers of MUT genes expression [20–22]. The increasing information gathered about MUT gene expression has allowed some researchers to develop methanol-free expression systems based on MUT machinery [19, 23, 24]. Such systems do not need methanol to trigger MUT genes because their TF genes have been derepressed by genetic engineering.

Some researchers have focused on the relationship between heterologous gene dosage and protein production rate. As previously reported, in P_*AOX1*_-driven expression systems, gene dosage and protein production are usually positively correlated, albeit with a relatively small number of copies (2 or 3) only [25–28]. However, producer clones integrating high gene of interest (GOI) expression cassettes are often subject to folding and secretion restrictions that result in oxidative stress in the endoplasmic reticulum, thereby having a direct impact on protein production. Also, producer clones containing large numbers of copies have been found to possess a limited transcription efficiency [25, 28]. According to Cámara et al. [28], the main limitation in strains with a large number of GOI copies occurs at the transcriptional level rather than in folding or secretion processes. Interestingly, both P_*AOX1*_-driven *Rhizopus oryzae* lipase (*ROL*) gene and MUT genes (*AOX1* included) have been found to be downregulated in clones with a large number of GOI copies, a limitation that results in decreased Rol production and methanol accumulation in chemostat cultivations.

Furthermore, specific growth rate (*µ*) has been confirmed as a key parameter that affects the specific protein production rate (*q*_*p*_). To date, many attempts to correlate both parameters have been successfully made. Thus, a positive relationship between them was observed when producing different proteins under the P_*GAP*_ [29–31] and P_*AOX1*_ control [32]. As the *P. pastoris* endogenous genes controlled by these promoters play crucial roles in glycolysis and methanol metabolism, respectively, the protein production driven by these expression systems are coupled to cell growth. By contrast, other authors point out the presence of a maximum in the *q*_*p*_–*µ* curve. Thus, Prielhofer et al. [33], observed a bell-shaped relationship between *q*_*p*_ and *µ* when expressing i-bodies under the control of an improved glucose-repressible P_*GTH1*_ promoter. These results led them to devise an optimized bioprocess strategy based on a stepwise decrease in *µ* during their fed-batch experiments. Canales et al. [34] studied the effect of glycerol:methanol mixtures in the chemostat feeding stream and the specific growth rate on Rol production under P_*AOX1*_ promoter. They found *µ* to be much more influential on *q*_*p*_ than was the methanol fraction in the feeding.

In this work, the integrated effect of *µ* and gene dosage on *AOX1* gene regulation and production kinetics of *Candida rugosa* lipase 1 (Crl1) driven by P_*AOX1*_ in *P. pastoris* was studied for designing a rational approach to optimize the operating conditions. For this purpose, a single-copy clone (SCC) and a multi-copy clone (MCC) were both cultivated under chemostat conditions to establish the relationship between *µ*, *CRL1* relative transcript levels (RTL) and *q*_*p*_. This correlation has allowed determining the operational strategy that maximizes Crl1 production. Additionally, transcriptional analyses of two key genes involved in methanol metabolism—*AOX1* and *MIT1*—were used in order to establish whether this pathway might be limited under specific conditions. Finally, Fed-batch cultivations were used to confirm the *q*_*p*_–*µ* profile pattern observed with chemostat cultivations to validate this experimental platform for the standard industrial operation mode used in *P. pastoris* cell factory.

## Results and discussion

### Effect of increasing *CRL1* gene dosage on culture physiological state

Increasing the dosage of heterologous genes is known to affect homeostasis in *P. pastoris* cultivations through restrictions in protein processing [35, 36]. Also, P_*AOX1*_-driven expression systems have been found to exhibit attenuated MUT gene expression [28], thereby affecting the methanol uptake rate (*q*_*s*_) of producer strains and potentially reducing their ability to grow [27, 36, 37].

**Fig. 1:**
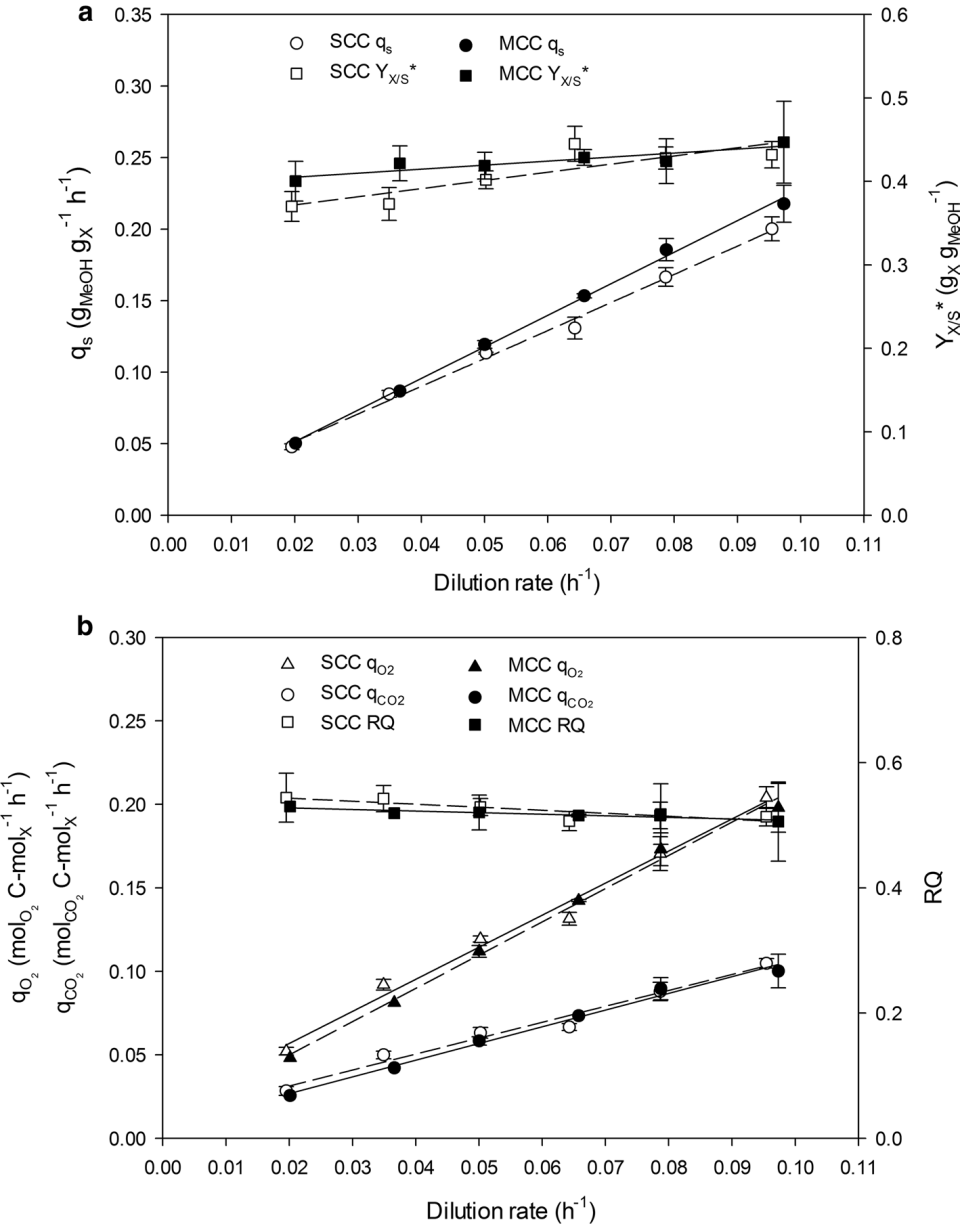
*Pichia pastoris* physiological response to an increase in *CRL1* gene dosage in chemostat cultivations. **a** Specific methanol consumption rate (*q*_*s*_), overall biomass-to-substrate yield (*Y*_*X*/S_*). **b** Specific oxygen uptake rate ($q_{{O_{2} }}$), specific carbon dioxide production rate ($q_{{CO_{2} }}$) and respiratory quotient (RQ). Error bars represent the standard deviation of two biological replicates

Figure 1a shows the variation of the specific substrate uptake rate (*q*_*s*_) and overall biomass-to-substrate yield (*Y*_*X*/S_*) over a wide range of dilution rates (*D*) (0.020–0.095 h^−1^) in chemostat cultivations of SCC and MCC. No methanol accumulation was observed under any conditions, but no *D* values above 0.095 h^−1^ were used in order to avoid washout. In addition, the carbon and electron balances were checked and deviations prior to data reconciliation found to be less than 5%. With both clones, *q*_*s*_ increased linearly across the *D* range, and *q*_*s*_ values at equivalent *D* values were rather similar for both clones. As a result, intrinsic substrate-to-biomass yield (*Y*_*S/X*_), and their respective maintenance coefficients (*m*_*s*_), were very similar (Table 1). Interestingly, both clones had mean *Y*_*S*/*X*_ values around 2.2 g_MetOH_ g_X_^−1^. This value is similar to the yield for the wild-type strain [38] and a slightly lower than reported for an important number of recombinant protein producer strains, which *Y*_*S/X*_ ranges 2–3 g_MetOH_ g_X_^−1^. However, for the recombinant production of other target proteins *Y*_*S/X*_ can reach higher values [6]. For instance, *Y*_*S/X*_ reached in the production of Rol under the same expression system was twofold higher than those obtained in the present work [13]. About the value of *m*_*s*_ reached for both clones, no significant statistical differences were observed. Results fell within the range of values reported in the literature, from 0.007 to 0.042 g_MetOH_ g_X_^−1^ h^−1^.

**Table 1:** Intrinsic yields (*Y*_*i*/*X*_) and maintenance coefficients (*m*_*i*/*X*_) from biomass growth obtained from chemostat cultivations

	Single-copy clone	Multi-copy clone
*Y*_*S/X*_ (g_MeOH_ g_X_^−1^)	2.16 ± 0.08	2.21 ± 0.05
*m*_*s*_ (g_MeOH_ g_X_^−1^ h^−1^)	0.014 ± 0.005	0.007 ± 0.004
$Y_{{CO_{2} /X}}$ (${\text{mol}}_{{{\text{CO}}_{2} }}$ C-mol_X_^−1^)	0.95 ± 0.07	1.00 ± 0.05
$m_{{CO_{2} }}$ (${\text{mol}}_{{{\text{CO}}_{2} }}$ C-mol_X_^−1^ h^−1^)	0.012 ± 0.005	0.007 ± 0.003
$Y_{{O_{2} /X}}$ (${\text{mol}}_{{{\text{O}}_{2} }}$ C-mol_X_^−1^)	1.92 ± 0.11	1.99 ± 0.07
$m_{{O_{2} }}$ (${\text{mol}}_{{{\text{O}}_{2} }}$ C-mol_X_^−1^ h^−1^)	0.018 ± 0.007	0.010 ± 0.005

± Indicates standard error (SE) from regression analysis

Other factors affecting the physiological state of the cell factory are related to respiration parameters, such as the specific O_2_ uptake rate ($q_{{O_{2} }}$), the specific CO_2_ production rate ($q_{{CO_{2} }}$), the corresponding intrinsic yields (*Y*_*i*/*X*_) and maintenance coefficients (*m*_*i*/*X*_), and the respiratory quotient (RQ). As can be seen from Fig. 1b and Table 1, these factors exhibited identical trends and similar values across the *D* range regardless of the gene dosage. Similar $Y_{{O_{2} /X}}$ were obtained for the human serum albumin [39], antibody production [40] and human chymotrypsinogen B [41] between 0.08 and 0.09 ${\text{mol}}_{{{\text{O}}_{2} }}$ g_X_^−1^. However highest values around 0.15 ${\text{mol}}_{{{\text{O}}_{2} }}$ g_X_^−1^ were obtained for *Rhizopus oryzae* lipase [13]. The higher values of $Y_{{O_{2} /X}}$ are directly related with the lower *Y*_X/S_* obtained. The results suggest that the values of the different yields are a function of the target protein expressed.

**Fig. 2:**
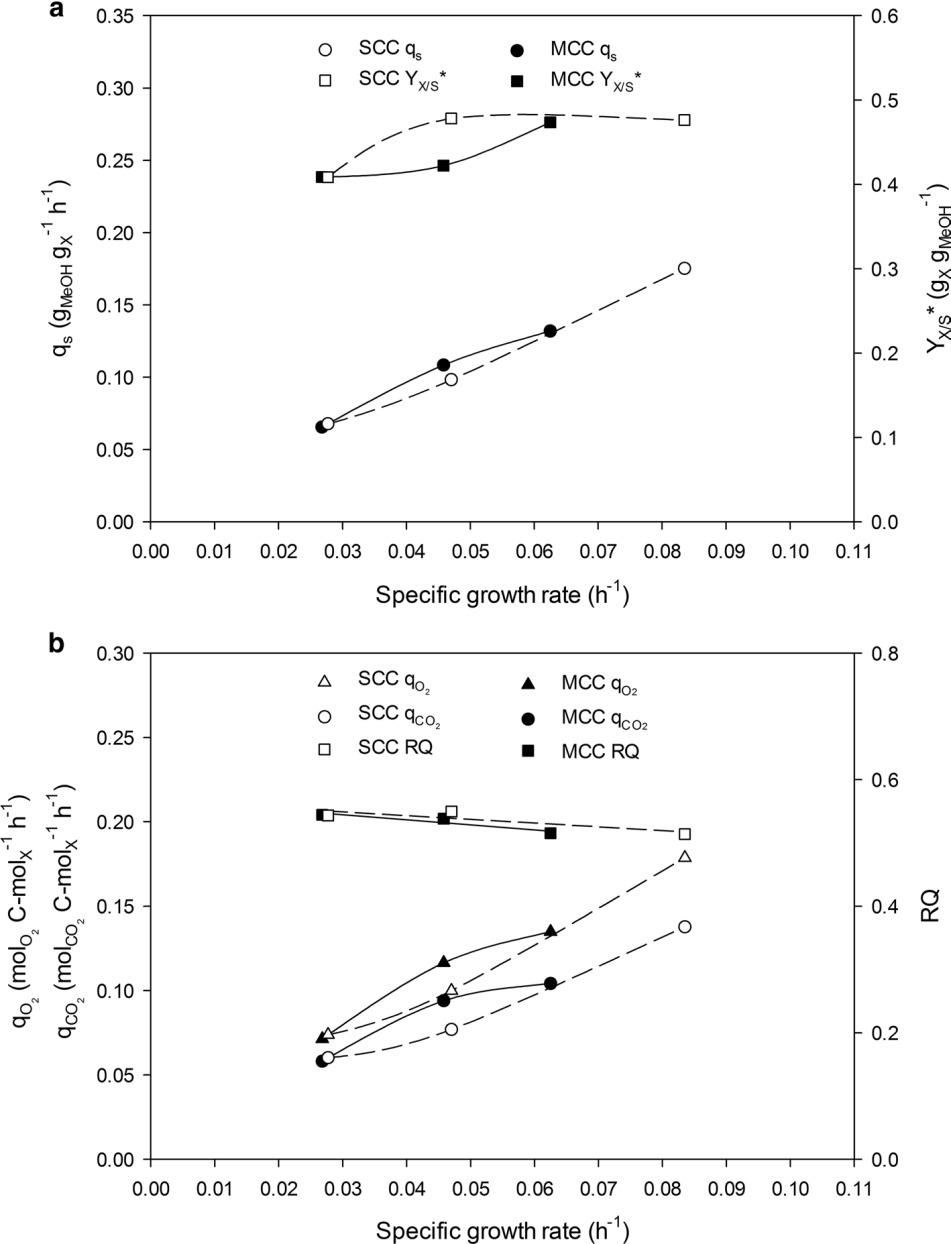
*Pichia pastoris* physiological response to an increase in *CRL1* gene dosage in fed-batch (FB) cultivations. **a** Specific methanol consumption rate (*q*_*s*_), overall biomass-to-substrate yield (*Y*_*X*/S_*). **b** Specific oxygen uptake rate ($q_{{O_{2} }}$), specific carbon dioxide production rate ($q_{{CO_{2} }}$) and respiratory quotient (RQ)

The fact that the major macrokinetic parameters related to the physiological status were very similar for both clones suggests that increasing the *CRL1* cassette dosage from one to three in *P. pastoris* genome had no effect on the physiological performance of the yeast in chemostat cultivations. On the other hand, a marked influence of the operational mode on such parameters was observed in FB cultivations of both clones. As can be seen in Fig. 2a, b, the evolution of *q*_*s*_, $q_{{CO_{2} }}$ and $q_{{O_{2} }}$ across the range of *µ* tested were different for both clones. These rates followed a nearly linear trend for SCC, similar to the behaviour observed on chemostat cultivations. In contrast, a saturated curve trend was obtained for MCC, thus indicating that the higher MCC metabolic burden caused by an increase heterologous gene dosage affects the clones capabilities for both methanol and O_2_ consumption, as well as CO_2_ production in this operational mode. Consequently, accumulation of methanol on MCC in fed-batch cultivation was observed at the highest *µ* tested (0.08 h^−1^). Thus, maximum *µ* tested for MCC was decreased to 0.065 h^−1^ in order to maintain carbon-limiting conditions. Although significant differences in $q_{{CO_{2} }}$ and $q_{{O_{2} }}$ were observed, RQ was quite similar in both operational modes irrespective of *µ*.

### Relationship between Mit1 limitation and a decreased *AOX1* relative expression (RE)

**Fig. 3:**
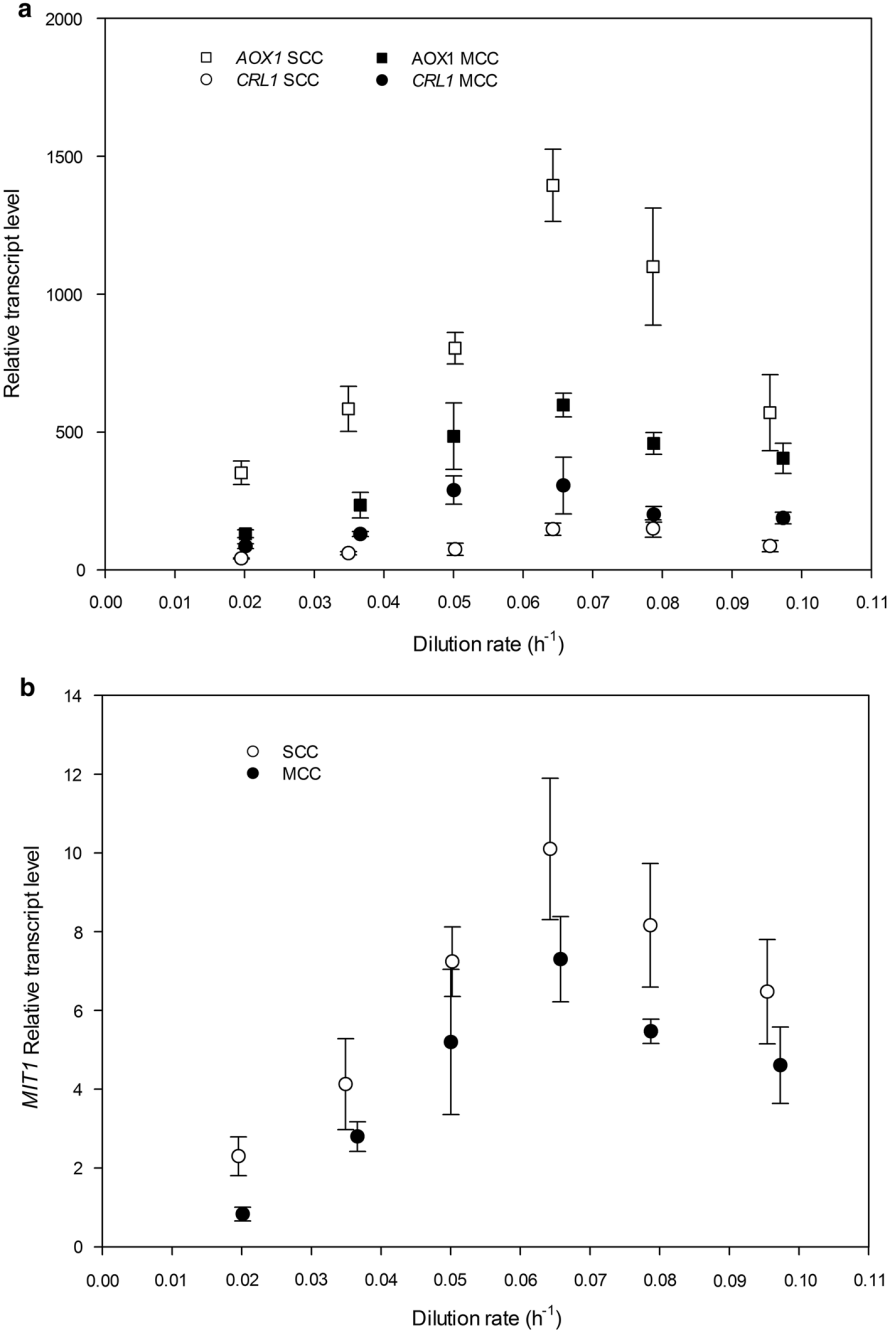
Influence of the dilution rate on relative gene transcription levels (RTLs) in chemostat cultivations. **a** Genes *AOX1* and *CRL1*. **b** Gene *MIT1*. *MTH1* was used as housekeeping gene for the analysis. Error bars represent the standard deviation of two biological replicates

The induction of strains with multiple copies of a P_*AOX1*_-driven heterologous gene with methanol has been reported to result in transcriptional limitation of MUT genes [28]. Therefore, transcriptional analysis of *AOX1*, *CRL1* and the methanol-induced transcription factor 1 (*MIT1*) genes were performed in order to examine their impact on the first step of methanol metabolism (see Fig. 3a, b).

These analyses were only carried out in chemostat cultivations, which were performed in steady-state conditions. As can be seen from Fig. 3a, and consistent with previous results [37], *AOX1* gene expression was in average twofold higher in SCC than it was in MCC, whichever the dilution rate. It indicates that the resources needed to trigger transcription of P_*AOX1*_-driven genes may be shared among them—heterologous gene cassettes and the endogenous *AOX1* gene included.

This phenomenon was further studied by analyzing transcriptional levels of *MIT1*, a crucial TF for P_*AOX1*_ induction [21], in both clones. As can be seen from Fig. 3b, *MIT1* relative transcript levels (RTL) were not significantly different comparing both clones across the *D* range tested, except for two dilution rates (0.02 h^−1^ and 0.08 h^−1^). Therefore, as expected, increasing heterologous gene dosage has not led to a proportional increase in *MIT1* transcription rate. One should therefore hypothesize that the Mit1 pool is a limited resource, all the genes whose expression depends on P_*AOX1*_ promoter would compete with one another for this TF—and hence for being transcribed. Consequently, the *AOX1* gene was less strongly expressed in MCC than it was in SCC owing to competition with three *CRL1* copies for the equivalent Mit1 resources. This hypothesis of Mit1 limitation is reinforced by the work of Cámara et al. [42] where the overexpression of Mit1 is enough to reverse the transcriptional limitation derived from increasing heterologous gene dosage. Moreover, deregulating the expression of some MUT-related TFs increased protein production driven by the P_*AOX1*_ expression system even in absence of methanol [19, 24, 42, 43].

Although these insights were previously obtained from a heterologous gene dosage comparison, no similar studies had examined a potential correlation of MUT-related genes RTL with the *µ*. A positive proportional relationship between *µ*, *MIT1* RTL and P_*AOX1*_-driven transcription rate should be expected since the more methanol was fed to the culture, the greater was the amount of *AOX1* enzyme needed to consume it. However, as can be seen in Fig. 3a, b, the correlation of the MUT-related genes RTL with *D* was bell-shaped for both clones, which suggests a close relationship between P_*AOX1*_-driven genes expression and *MIT1*. Further research at transcriptional level would be needed to elucidate why *P. pastoris* decreases its methanol consumption resources when it approaches its maximum specific growth rate, *µ*_*max*_.

Overall, the previous results show that the *MIT1* RTL is governed by *µ*, affecting the transcription rate of P_*AOX1*_-driven genes. One could hypothesize that a similar phenomenon could also take place for to other MUT-related TFs such as Mxr1 and Prm1, since the regulation of their expression in presence of methanol must be coupled [21]. Although *AOX1* RTLs were low in MCC relative to SCC, *AOX1* was expressed strongly enough to produce the minimum amount needed to catabolize all methanol fed to chemostat cultivations. However, as noted in the previous section, growing FB cultivations of MCC at the maximum *µ* level reached by SCC (0.08 h^−1^) led to methanol accumulation during early stages of the feeding phase.

### Influence of operational mode on production-related parameters

**Fig. 4:**
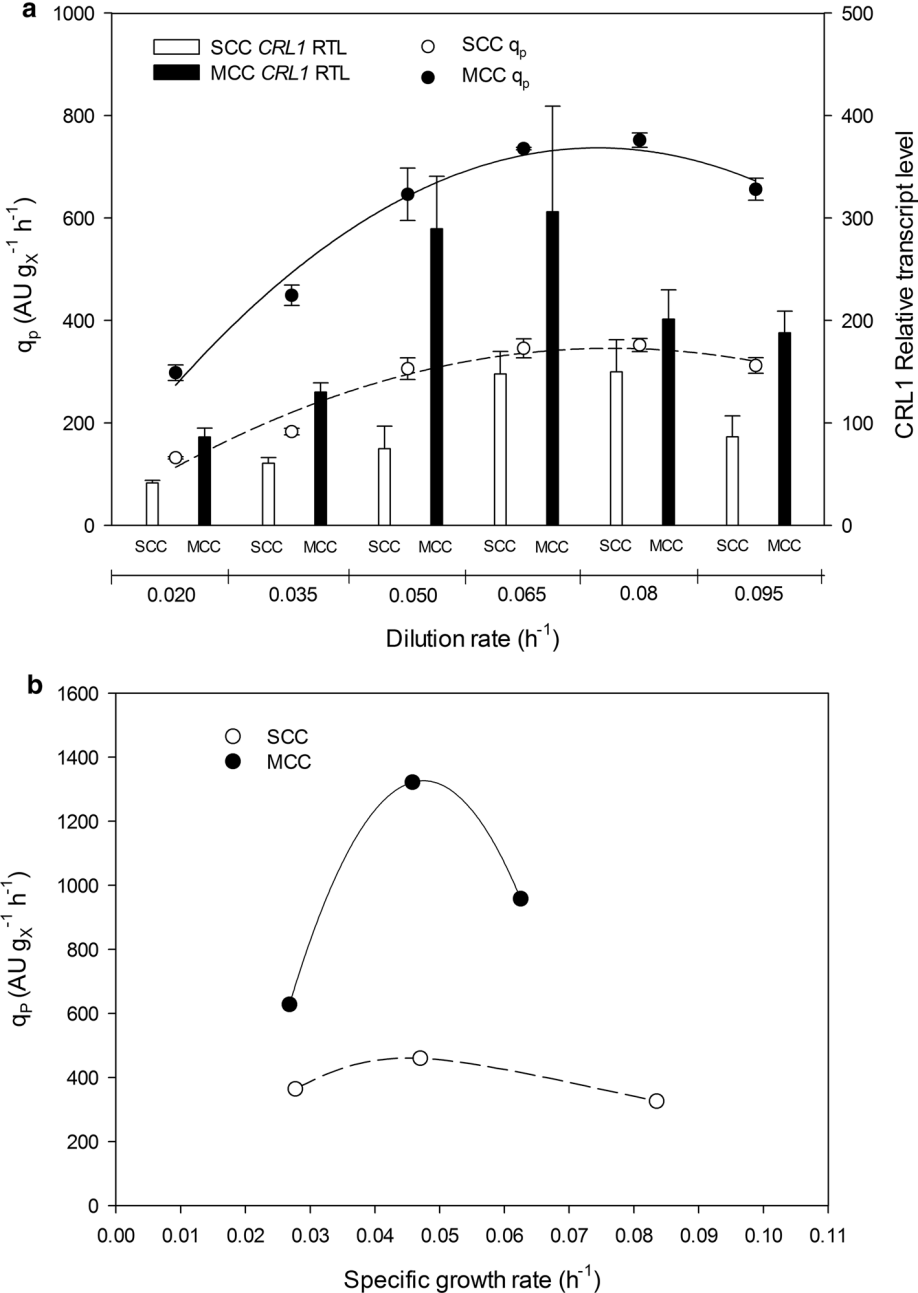
Comparison of SCC and MCC Crl1 production kinetics and its relationship with *CRL1* relative transcription levels. The specific Crl1 production rate (*q*_*p*_) was calculated for chemostat (**a**) and fed-batch cultivations (**b**). *CRL1* transcriptional analyses were also done on chemostat cultivations (**a**). *MTH1* was used as housekeeping gene for *CRL1* RTL calculations. Error bars represent the standard deviation of two biological replicates

The primary aim of this work was to elucidate the Crl1 production kinetics for both clones in chemostat and FB cultivations. Furthermore, transcriptional analysis provided valuable information, which also should be related with both the growth and the recombinant protein production. Since Crl1 production in this cell factory is governed by the P_*AOX1*_ promoter, it was expected to be coupled to growth because the sole carbon source used was methanol [32]. However, *q*_*p*_ was not linearly related to *D* or *µ* in either operational mode. Rather, both chemostat and FB cultivations exhibited a bell-shaped trend in both clones, production being optimal at *D* = 0.08 h^−1^ in chemostat cultivations (Fig. 4a) and *µ* = 0.045 h^−1^ in FB cultivations (Fig. 4b). In FB cultivations, MCC gave a more pronounced bell-shape curve than did SCC. Therefore, MCC would require a more precise control of *µ* because a slight deviation from the optimal set-point would result in a marked decrease in *q*_*p*_. Consequently, the optimum differences in *µ* should be considered in designing bioprocesses for recombinant protein production.

**Fig. 5:**
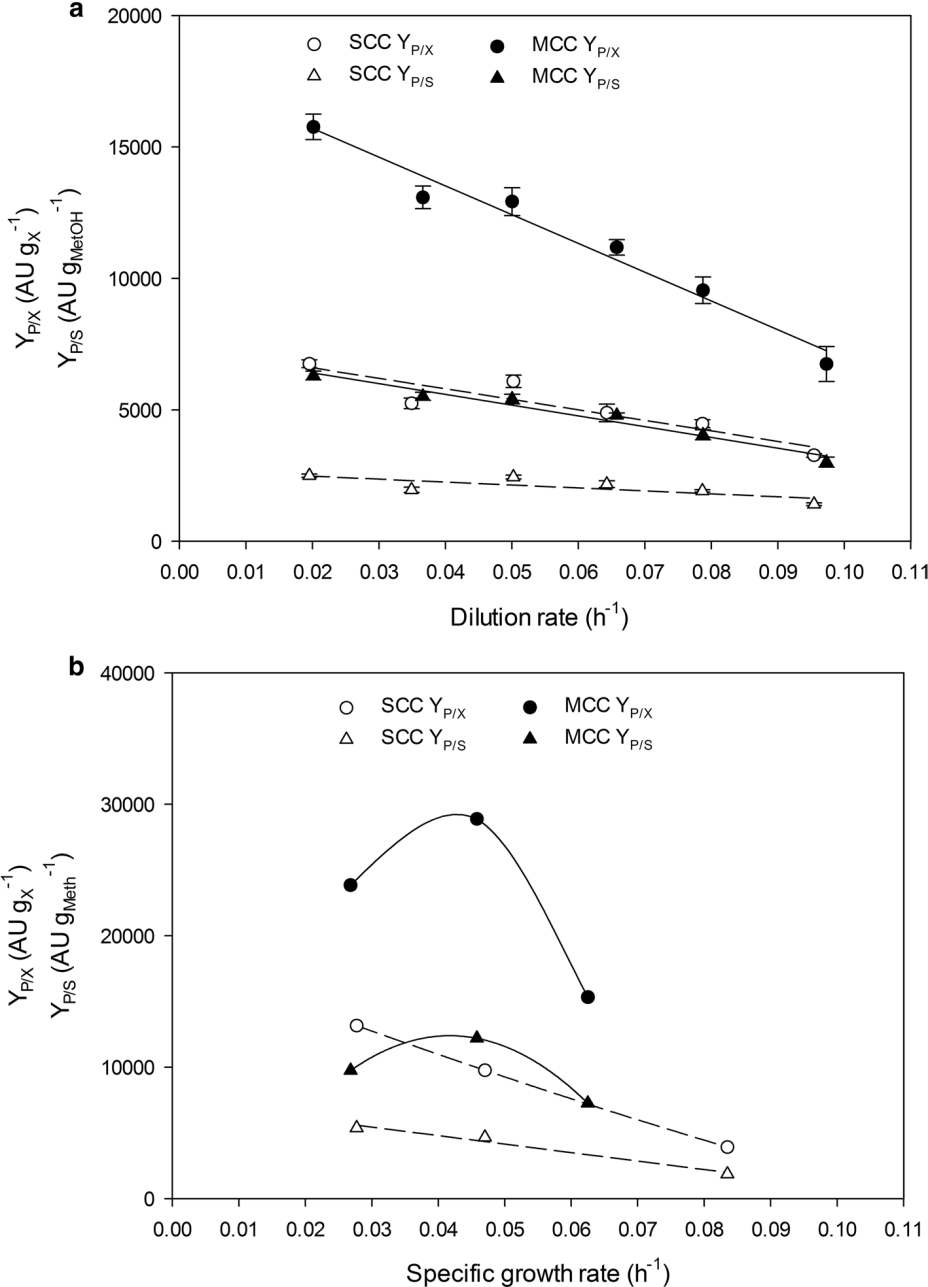
Comparison of SCC and MCC strain Crl1-related yields. Overall product-to-biomass yield (*Y*_*P*/*X*_) and product-to-substrate yield (*Y*_*P*/*S*_). **a** Chemostat cultivations. **b** Fed-batch cultivations. In **a**, error bars represent the standard deviation of two biological replicates

**Fig. 6:**
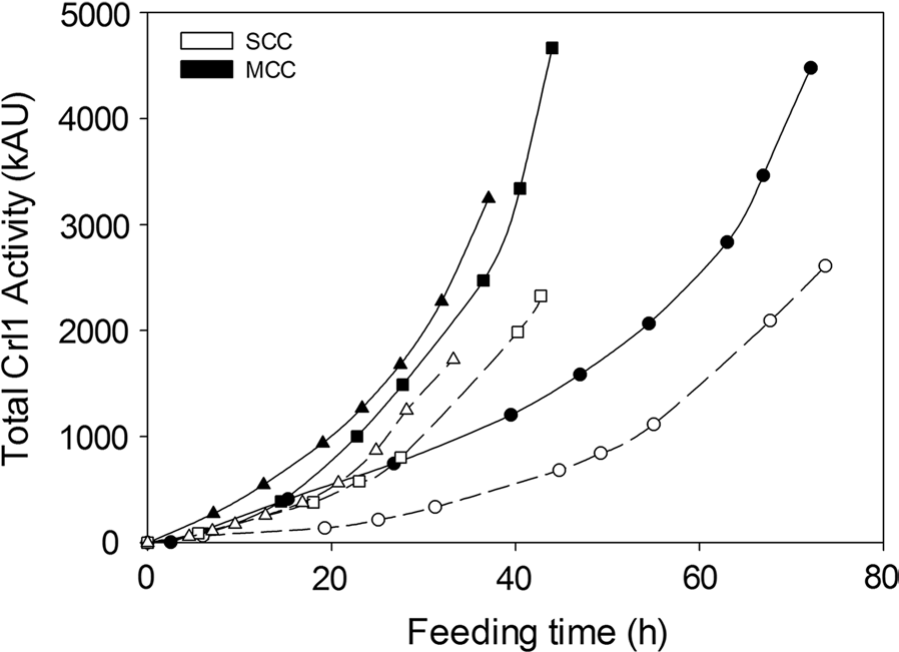
Crl1 production time evolution expressed as total activity units in fed-batch cultivations at different *µ*: (filled circle, open circle), 0.028 h^−1^; (filled square, open square), 0.047 h^−1^; (filled triangle), 0.063 h^−1^; (open triangle), 0.084 h^−1^

The overall product-to-biomass and product-to-substrate yield (*Y*_*P*/*X*_ and *Y*_*P*/*S*_, respectively) exhibited a linear decreasing trend in chemostat cultivations of both clones (Fig. 5a). Although SCC behaved identically in FB cultivations, the MCC exhibited a maximum value at an intermediate *µ* level (0.045 h^−1^; Fig. 5b). These yields are important inasmuch as they are closely related to product titer (Fig. 6), which is a parameter susceptible to be optimized in industry due to its influences on downstream processing costs. Therefore, MCC would be the strain of choice for optimum Crl1-related yields and titers when cultivated at intermediate *µ* values. Irrespective of gene dosage, *q*_*p*_ and product-related yields were more than twice greater in FB cultivations than they were in chemostat cultivations.

### Increasing *CRL1* gene dosage boosts protein production

Although a transcriptional limitation had been proved in at least two MUT-related genes, it was necessary to quantify to what extent the GOI transcription rate was affected by an increase in gene dosage, and hence how it influenced *q*_*p*_.

**Fig. 7:**
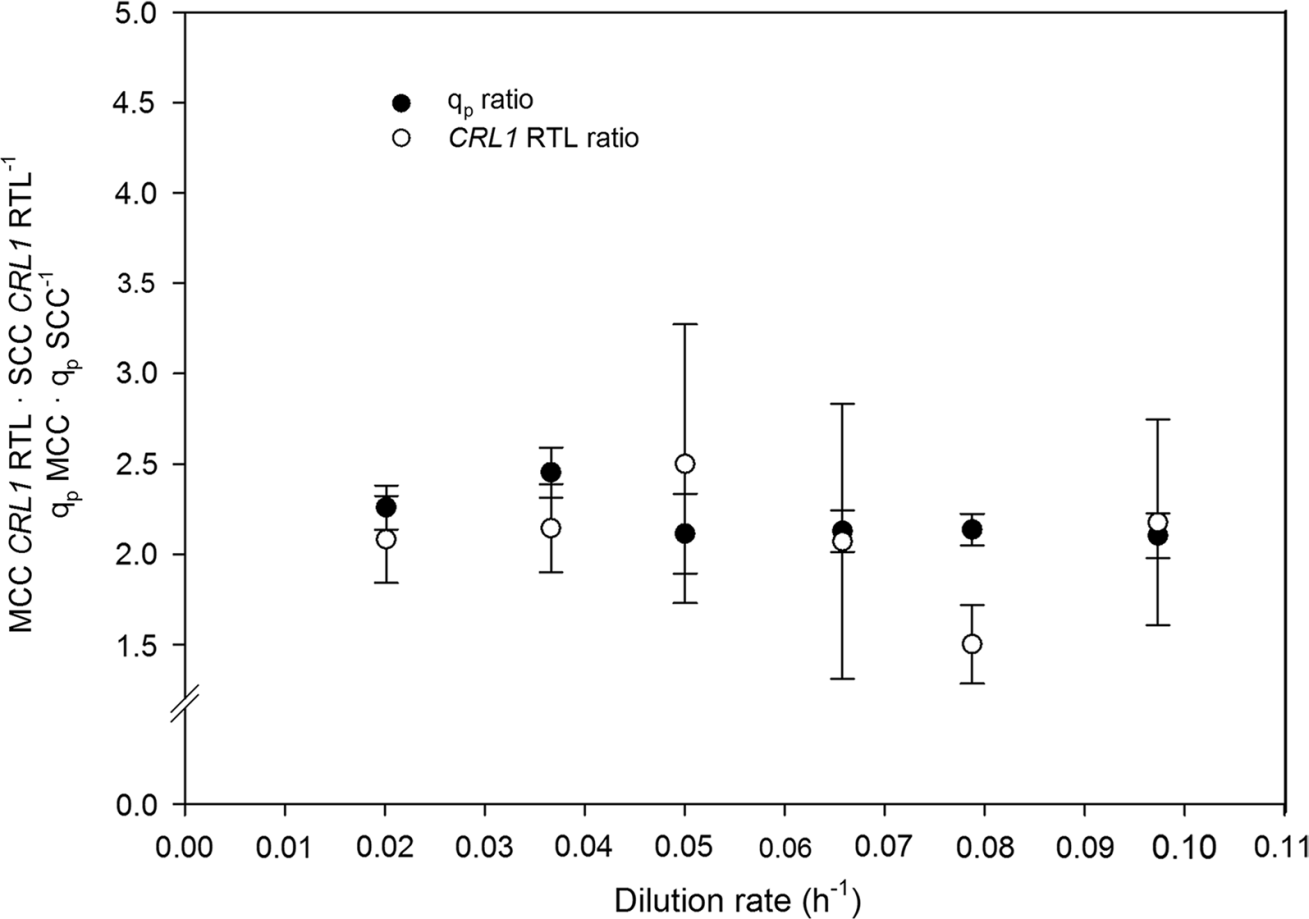
Effect of dilution rate on the *CRL1* relative transcription level and specific production rate ratios between MCC and SCC. Error bars represent the standard deviation of *q*_*p*_ and RTL ratios

In chemostat cultivations, *CRL1* RTL was on average 2.2 fold higher in MCC than in SCC across the *D* range (Fig. 7), which was also reflected on *q*_*p*_ raises between 2.1 and 2.4 (Table 2). This correlation between *CRL1* RTL and *q*_*p*_ ratios suggests the absence of a bottleneck in further protein processing-secretion steps, and hence in overall Crl1 production rate. This hypothesis was supported by conducting a transcriptional analysis of UPR-related genes such as *KAR2* and *HAC1*, the expression levels of which were rather constant across the *D* range (results not shown). Likewise, raises of other product-related parameters were also around 2 and 2.8-fold higher when comparing equivalent *D* conditions (Table 2).

**Table 2:** Comparison of Crl1 production-related parameters for chemostat cultivations

	Single copy clone						Multi copy clone					
*D* (h^−1^)	0.019	0.035	0.050	0.064	0.079	0.095	0.020	0.036	0.050	0.066	0.079	0.097
Product titer (AU mL^−1^)	124	101	118	107	93	68	321	267	274	243	212	155
Product titer ratio	–	–	–	–	–	–	2.59	2.64	2.32	2.27	2.28	2.28
*q*_*P*_ (AU g_X_^−1^ h^−1^)	132	183	306	346	352	312	297	449	646	735	751	656
*q*_*P*_ ratio	–	–	–	–	–	–	2.25	2.45	2.11	2.12	2.13	2.10
*Q*_*P*_ (kAU L^−1^ h^−1^)	2.43	3.51	5.91	7.59	7.31	6.51	6.46	9.81	13.72	16.02	16.71	15.15
*Q*_*P*_ ratio	–	–	–	–	–	–	2.66	2.79	2.32	2.11	2.29	2.33
*Y*_*P/S*_ (kAU g_S_^−1^)	2.50	1.96	2.45	2.17	1.92	1.41	6.31	5.52	5.41	4.79	4.05	3.01
*Y*_*P/S*_ ratio	–	–	–	–	–	–	2.52	2.8	2.21	2.21	2.11	2.13
*Y*_*P/X*_ (kAU g_X_^−1^)	6.76	5.25	6.09	4.88	4.48	3.27	15.77	13.08	12.92	11.18	9.55	6.74
*Y*_*P/X*_ ratio	–	–	–	–	–	–	2.33	2.49	2.12	2.29	2.13	2.06

The ratios between the MCC and SCC were calculated by dividing the MCC parameter values to SCC ones at similar dilution rate (*D*)

The improvement of product-related parameters could be considered slightly lower than expected, since MCC harbor three *CRL1* expression cassette copies. However, as widely reported, increasing the heterologous GOI dosage in the genome need not lead to a proportional increase in protein production rates [25–28]. In this case, the low *CRL1* transcription efficiency—*CRL1* RTL was just 2.2-fold higher on MCC—is the responsible of the lower than expected product-related parameters values.

The increase in protein production derived from increasing the *CRL1* gene dosage in FB cultivations was similar to that in chemostat cultivations. In fed-batch cultures, however, the ratios between clones increased with increasing *µ* (Table 3). Thus, at low *µ* levels, product-related parameters such as titer, *q*_*p*_, *Q*_*p*_ (volumetric productivity) and product yields were about 1.8 fold higher in MCC than they were in SCC. At intermediate *µ* levels, the previous parameters were roughly 2.3 fold higher in MCC. At the highest *µ* level, however, the comparison was not reliable since the culture conditions were not equivalent. Specifically, the *µ* set-point used with MCC had to be adapted to avoid an eventual methanol accumulation.

**Table 3:** Comparison of Crl1 production-related parameters for fed-batch cultivations

	Single-copy clone			Multi-copy clone		
*µ* (h^−1^)	0.028	0.047	0.084	0.027	0.046	0.063
Product titer (AU mL^−1^)	769	660	261	1386	1542	1033
Product titer ratio	–	–	–	1.80	2.32	3.95
*q*_*P*_ (AU g_X_^−1^ h^−1^)	364	460	326	628	1322	958
*q*_*P*_ ratio	–	–	–	1.73	2.25	2.93
*Q*_*P*_ (kAU L^−1^ h^−1^)	7.87	10.89	5.53	14.66	21.81	18.01
*Q*_*P*_ ratio	–	–	–	1.86	2.00	3.26
*Y*_*P/S*_ (kAU g_S_^−1^)	5.37	4.67	1.86	9.74	12.19	7.25
*Y*_*P/S*_ ratio	–	–	–	1.81	2.74	3.90
*Y*_*P/X*_ (kAU g_X_^−1^)	13.16	9.76	3.91	23.84	28.88	15.32
*Y*_*P/X*_ ratio	–	–	–	1.81	2.56	3.91

The ratios between the MCC and SCC were calculated by dividing the MCC parameter values to SCC ones at similar specific growth rate (*µ*)

Overall, increasing the *CRL1* gene dosage resulted in increased protein production in both chemostat and FB cultivations.

### Transcriptional efficiency differences between *AOX1* and *CRL1* genes, and their impact on Crl1 production

**Fig. 8:**
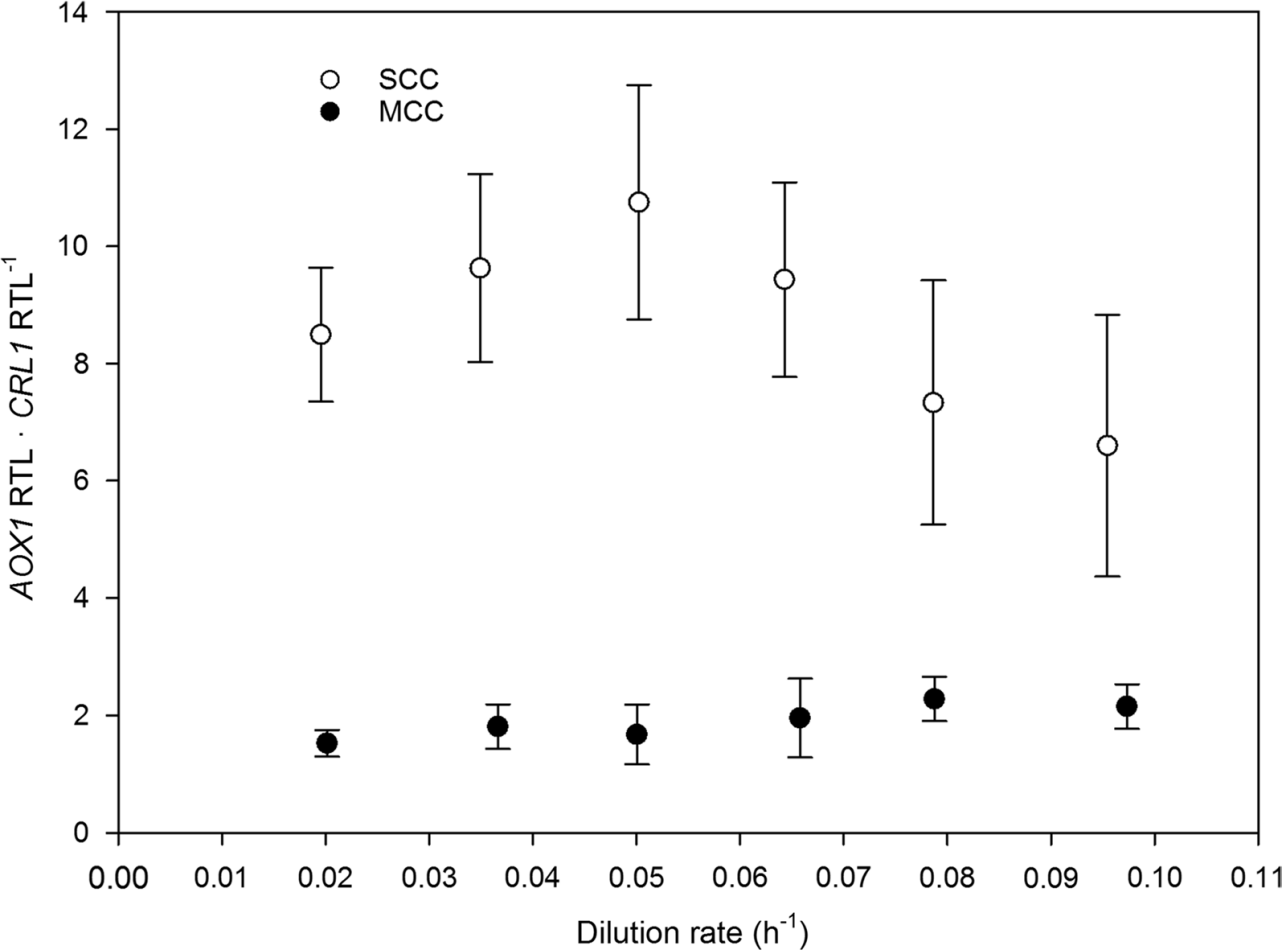
Effect of dilution rate on the *AOX1*–*CRL1* relative transcription level ratio between MCC and SCC Error bars represent the standard deviation of RTL ratios

Regarding the balance between the transcription levels of the *AOX1* and the *CRL1* genes, being both P_*AOX1*_-driven, an unexpected ratio between *AOX1* RTL and *CRL1* RTL was found in chemostat cultivations. As can be seen from Fig. 8, *AOX1* RTL was considerably greater than *CRL1* RTL in SCC, the difference ranging from 11 times at low *D* values to 6 times at the highest one. Even in MCC, which harbor three *CRL1* expression cassettes—versus only one of *AOX1*—, *AOX1* RTL exceeded clearly the *CRL1* RTL. The amount of mRNA a given gene contains is known to depend on the balance between transcription rate and mRNA decay [44]. Therefore, since both coding sequences were flanked by the same promoter (P_*AOX1*_) and transcription terminator (*AOX1*) here, one should expect the transcription rate to be similar. Hence, the differences in mRNA between *CRL1* and *AOX1* might be related with mRNA stability and hence with mRNA degradation.

As shown in Fig. 7, *CRL1* RTL was closely correlated with *q*_*p*_ as a result of the absence of stacks in folding, trafficking and secretion processes. The *AOX1* RTL/*CRL1* RTL ratio was thus a crucial parameter. As noted earlier, the pool of MUT-related TFs that can be shared by all the P_*AOX1*_-driven genes is expected to be limited. As a result, increasing the number of *CRL1* cassettes in the genome should gradually increase the ratio up to a point where *AOX1* expression would not be enough to consume all the methanol fed in the culture. This hypothesis was confirmed in those cases where methanol accumulation was substantial. In this expression system, increasing the *CRL1* gene dosage to three expression cassettes (MCC) reduced the *AOX1*-to-*CRL1* RTL ratio from 10 to 7 in SCC to 2 in MCC; as a result, *q*_*p*_ was increased by a factor of 1.7–3 without appreciably affecting the ability to metabolize methanol fed to the culture. Therefore, the SCC expression system could be considered inefficient for producing Crl1 because the cell factory *P. pastoris* expresses higher levels of *AOX1* than the essentially needed, which is detrimental to Crl1 production.

Further increasing the number of *CRL1* cassettes is therefore the way of identifying the optimum *AOX1* RTL/*CRL1* RTL ratio for maximal Crl1 production without detracting from the physiological capabilities of the yeast. However, potential bottlenecks arising from an increased protein production should be considered.

## Conclusions

In this work, the influence of the heterologous gene dosage was used to expose the high importance of *µ* on the transcription of MUT genes, production kinetics and culture physiological status. Increasing the *CRL1* gene dosage was found not to affect the clone capabilities in terms of methanol and oxygen uptake rate (*q*_*s*_ and $q_{{O_{2} }}$, respectively), nor the carbon dioxide production rate ($q_{{CO_{2} }}$), at least at the macrokinetic level in chemostat cultivations. On the other hand, a significant effect on these parameters was observed in fed-batch cultivations for MCC. Specifically, a saturation pattern was observed in all physiology-related macrokinetic parameters across the *µ* range studied. This result departs from the expected linear trends, which were indeed observed in chemostat cultivations. Also, the influence of the operational mode on the physiological status was significantly higher in MCC than in SCC.

According to the results presented, *µ* seemingly determines the expression of *MIT1*, which have a key role in triggering transcription of P_*AOX1*_-driven genes, thus influencing the amount of protein of interest that is produced at the end of the process. Also, increasing the number of *CRL1* expression cassettes integrated in the genome from one to three in *P. pastoris* effectively boosted production without significantly altering the physiological status of the yeast. Furthermore, since increasing the *CRL1* dosage strongly reduced *AOX1* expression, one could hypothesize an eventual limitation of the TF *MIT1* pool, which is supported by our results. Finally, the strong correlation between *CRL1* RTL and specific *CRL1* production rate in both clones suggests the absence of bottlenecks in protein processing processes for this particular expression system in chemostat cultivations.

As shown in the present work, the operational mode used considerably influences product-related parameters such as *Y*_*P*/*X*_ and *q*_*p*_, which were twice greater in the fed-batch cultivations than they were in the chemostat cultivations at identical specific growth rates for Crl1 production in this expression system.

Interestingly, by means of analysing both P_*AOX1*_-driven expression, we prove that the transcription efficiency is an important factor to study in any case. In our case, despite being flanked by same promoter and terminator, the expression of *AOX1* and *CRL1* genes were highly different. Therefore, when using endogenous expression systems to produce a protein—like P_*AOX1*_, the most important factor to analyse is not only the gene dosage but the expression difference between the endogenous and the heterologous gene.

The outcome of these experiments expects to provide a wealth of knowledge for designing a rational approach to optimizing the operating conditions. Although the production patterns are expected to be similar for different proteins of interest to be expressed, the outcome usually depends on the particular expression regulation system as well as the target protein. Therefore, similar experiments should be conducted in each case, not only to maximize production rates, but also to identify the most suitable conditions for testing other strains with industrial potential.

## Materials and methods

### Plasmid construction and strain generation

Recombinant strains of *P. pastoris* expressing *CRL1* gene under the regulation of P_*AOX1*_ were constructed by using the pPICZαA plasmid (Invitrogen, Carlsbad, CA, US) assembled with the codon-optimized synthetic open reading frame (ORF) encoding the *CRL1* gene sequence (GeneScript, Piscataway, NJ, USA). Then *P. pastoris* X-33 cells (Invitrogen, Carlsbad, CA, US) were transformed with chimeric vector under the conditions described elsewhere [42]. Therefore, two clones having one and three *CRL1* expression cassette copies, respectively, were selected and used for this study.

### Gene dosage determination

The number of *CRL1* expression cassettes integrated into the genome was determined by droplet digital PCR (ddPCR) according to Cámara et al. [45]. The actin gene (*ACT1*) was selected as a housekeeping gene for the analysis. The specific primers used are presented in Additional file 1.

### Total RNA extraction

Chemostat samples for RNA isolation were collected according to Landes et al. [46]. Pellets from 1 mL culture broth samples were resuspended in 1 mL of TRIzol™ reagent (Waltham, Massachusetts, USA) and lysed with glass beads (425–600 µm, Sigma-Aldrich, St. Louis, MO, USA) for mechanical disruption. Cell lysis was attempted by alternating cycles of 30-s of vortexing and freezing. All further steps were performed according to the manufacturer’s instructions.

RNA integrity was checked by agarose electrophoresis and the RNA concentration determined from Nanodrop measurements on an instrument from Thermo Scientific™ (Waltham, MA, US).

### Synthesis of cDNA and determination of transcriptional levels

cDNA was synthetized with the iScript™ cDNA Synthesis kit (Bio-Rad, Hercules, CA, USA), following the manufacturer’s instructions. For qPCR, a set of primers were designed for specific target cDNA. The set of selected genes comprised *CRL1* (heterologous gene); *AOX1*, the alcohol oxidase 1 native gene; and *MIT1*, which codifies a key transcription factor of the methanol-induced transcription. Furthermore, *KAR2* and *HAC1*, two genes involved in the unfolded protein response (UPR), were also analyzed.

For qPCR, reactions were done with SYBR™ Select Master Mix (Thermo Scientific™ Waltham, MA, US). Additionally, and as suggested by the manufacturer, to assure the maximum accuracy the reaction mixes were made by EpMotion^®^ 5075 robot (Eppendorf, Germany).

The amplification program was run on a QuantStudio 12 K Flex Real-Timer from Thermo Scientific™ (Waltham, MA, US), following the manufacturer’s instructions. The annealing extension temperature was set at 57.4 °C. Relative transcript levels (RTLs) were determined by using *MTH1* as a housekeeping gene as it shows basal expression across the conditions tested.

### Chemostat cultivation

Chemostat cultivation of the two clones were run in duplicate in a 2 L Biostat B plus Bioreactor (Sartorius Stedim, Goettingen, Germany) according to García Ortega et al. [30]. The medium composition was the same except that glucose was replaced with methanol as sole carbon source at a final concentration of 50 g L^−1^ on feeding. A wide range of dilution rates was covered. Specifically, it was tested the following dilution rates: 0.020 h^−1^, 0.035 h^−1^, 0.050 h^−1^, 0.065 h^−1^, 0.080 h^−1^, 0.095 h^−1^. Under each set of conditions, samples were obtained after five residence times. To ensure that a steady state was reached, samples were analyzed from the third residence time, to check the stability of the parameters of interest.

### Fed-batch cultivation

Both clones were also cultivated in the fed-batch mode, using a 5 L Biostat B Bioreactor (Sartorius Stedim, Goettingen, Germany) at different specific growth rates from 0.030 to 0.08 h^−1^ for SCC and 0.030 to 0.065 h^−1^ for MCC. An exponential pre-programming feeding rate was performed to maintain the specific growth rate constant at the selected set-point. All cultivations were grown under carbon-limiting conditions. The procedure is described in detail elsewhere [29]—by exception, the glucose/glycerol pair was replaced with a methanol concentration of 400 g L^−1^ in the feed.

### Biomass determination as dry cell weight (DCW)

Biomass concentrations were measured as DCW values as described elsewhere [47]. The relative standard deviation (RSD) of the measurements was about 3%.

### Quantification of the carbon source and byproducts

The concentration of the different carbon sources used in the batch (glycerol), and chemostat and fed-batch cultivations (methanol), and the potential fermentation byproducts, were all determined by HPLC. The column and program used are described elsewhere [48]. RSD was invariably less than 1%.

### Off-gas analyses

A *BlueInOne Cell* gas analyzer (BlueSens, Herten, Germany) was used with both chemostat and fed-batch cultivations. The CO_2_ and O_2_ mol fractions were recorded online with provision for off-gas pressure and humidity. The data thus obtained were used to calculate the oxygen uptake rate (OUR), carbon dioxide evolution rate (CER), specific rates ($q_{{O_{2} }}$ and $q_{{CO_{2} }}$) and respiratory quotient (RQ). RSD was less than 5% in all cases.

### Lipolytic activity

An enzymatic *p*-nitrophenyl butyrate (*p*NPB) based assay was selected to determine Crl1 activity by using a procedure described elsewhere [49] albeit with slight modifications. Thus, 20 μL volumes of the samples were mixed with 980 μL of reaction buffer, which contained 1 mM *p*NPB, 50 mM phosphate buffer (pH 7) and 4% (v/v) acetone. The absorbance at 348 nm was monitored at 30 °C by using a *Specord 200 Plus* spectrophotometer from Analytic Jena (Jena, Germany). One activity unit was defined as the amount of enzyme needed to release 1 mmol of *p*-nitrophenol per minute under assay conditions. RSD was less than 1%.

### Process parameters determination, consistency checking and data reconciliation

#### Mass balance and stoichiometric equations

All equations derived from the mass balances used to calculate yields and rates in the chemostat [30] and fed-batch experiments [13] can be found elsewhere. The mean elemental biomass composition CH_1.78_ O_0.62_ N_0.18_ S_0.006_ with an ash content of 9% was determined as previously reported [47]. The carbon and electron balances were checked and less than 5% of deviation observed prior to reconciliation.

#### Consistency checking and data reconciliation

Measurement consistency was checked by using the standard test with carbon and electron balances as constraints. Both online and offline measurements allowed five key specific rates in the black-box process model to be calculated, namely: biomass generation (*μ*), glucose uptake rate (*q*_*s*_), product generation rate (*q*_*p*_), oxygen uptake rate ($q_{{O_{2} }}$) and carbon dioxide production rate ($q_{{CO_{2} }}$). The method used for this purpose is described in detail elsewhere [13].

## Supplementary information

**Additional file 1.** List of primers used for qPCR analyses.

## Data Availability

All data generated or analysed during this study are included in this published article and its additional files.
